# The Physiological Therapeutics of Masturbation

**Published:** 1900-09

**Authors:** C. A. F. Lindorme

**Affiliations:** Chicago, Ill.


### THE PHYSIOLOGICAL THERAPEUTICS OF MASTURBATION.

BY C. A. F. LINDORME, PH.D., M.D.,
CHICAGO, ILL.

Once, in a conversation I had with a masturbator who consulted me, I emphasized that of all he could do to disabuse himself, the main thing would be his determinate will to leave off.

"O, it is not my will that does it," he answered.

By the inflection of voice with which he uttered this remark I could tell that he thought himself well backed in his psychological theory. But unfortunately for him the downright philosophy which I owed to the study of Shakespeare made me a match for such metaphysical subterfuge, and when I, in return, addressed to him the question, "Well, whose will is it, if not yours?" all he saw fit to do was to smile.

Rudimentary metaphysicians, as there are so many among the speculating physiologists, teach that Napoleon was not at liberty to accomplish his conquests, but had to do that conquering. He did it on compulsion. Now, this is something against which Falstaff even revolts, and if we were to admit the philosophical burlesque as a controversy we might ask, whether, if Napoleon had been at liberty to do so, he would have stayed at home and nursed the sick in the hospitals, and sent Josephine to Austerlitz, and his brother Jerome to Jena? But, taken seriously, the point at issue is in the indistinctness of the personality. Those rudimentary metaphysicians look at the personality as on a clock which runs down as it is wound up. Now, it may be admitted that at birth there is in a child an intelligible character. But the environments, the outward influences, work as modifications, and not before he draws his last breath may man consider his personality completely consummate. The personality those rudimentary metaphysicians overlook is not a morphological unity, but a dynamical product, and changes sad and sorely at the least suspected moment:

"What devil was't
That thus has cozen'd you at hoodman-blind ?
Eyes without feeling, feeling without sight,
Ears without hands or eyes, smelling sans all,
Or but a sickly part of the true sense
Could not so mope."—Shakespeare.

Thought might be the determining factor in man's life, but for the will which rules it:

"Our wills and fates do so contrary run,
That our devices still are overthrown,
Our thoughts are ours, their ends none of our own."—Idem.

The human will is a region of exceedingly intricate psychology. But its foundation is easily understood, if we distinguish well its physiological anatomy. In the red blood-corpuscles we have the push, the active part of the will, in the nerve-cells the data of observation from which the will construes the motives of its action. The will is neither entirely in the corpuscles nor in the nerve-cells; it is in the relation of each to the other. By will, we understand generally, the tendency towards objects which as such cannot be gotten aware of except in an indistinct subjective feeling. A realization takes place by specification in the understanding. Here a special object is isolated more or less, as the most desirable one, and the tendency towards it is then in common language called the will. The true physiological relation is accordingly just the reverse of what the rudimentary metaphysicians would want to make it; substantially the will is altogether in the red blood-corpuscles, but its qualification in the outward world cannot receive except through the intermediacy of the brain.

The character of an individual must therefore be determined in proportion to this mutuality; it is constituted in this proportion of mutuality. If an individual is strong, consequently a good blood-producer, but, through encephalic paucity, lacking in cerebral moiety, creating a disproportion in the relation of red corpuscles to nerve-cells, such an individual will be subject to towering passions and may eventually become a dangerous member of society ; the motives of action he experiences in his encephalon will be tremendously imperious; as in an animal they will be powerful for want of choice: a tiger is not voracious from mere savagery, but for reason of cerebral restriction in forming motives for his will; the latter is accommodated with too scanty an encephalic outfit, intellect and above all imagination, to extend beyond the desire of feeding, and since in the wilderness the chances for this in sufficient extension are limited too, he is the brute of prey we know him. A negro committing rape on a white woman, all the danger of the crime fully known to him notwithstanding, is among men a case of the strictest analogy; in proportion to the promptings of his blood the cerebral outfit affording a control by dissuading, inhibitory motives are an altogether insufficient offset, and the man brings himself into a situation which is pitiable, but from a scientific standpoint by no means that which the traditional estimation, as sanctified by the law, makes it: The very revenge of the poor victim, if by Lynch or the regular Judge, a mere matter of form, is as it were an excuse of the committed crime; it is psychologically a duplication of the trespass of the negro, in every point of psychology, with the difference in the motives only, that of the negro being incontinence of sexuality, that of the other side incontinence of revengefulness, in the law-procedure with the additional feature of the slow, frigid cruelty of the headsman. The real cause is a remote one, the civic equality of the negro.

On the other hand, if a person is weak, consequently a poor blood-producer, but has a rich encephalic outfit, there will be a surfeit of motives, dissuading and inhibitory, and consequently very little determination, passion, push. In such a case it is likely that procrastination, indecision and in consequence of it neglect, ensues. There may be criminality. But there will no running of the criminal past himself. There will be more insidiousness of action. It is told of Coleridge, who was an instance of rich encephalic outfit and weak volitional substantiation, that, when walking in the park, he would stand still, indeterminate whether to take the right or the left side of the path. For lack of energy he took delight in abstraction in itself, and could be charmed by such stupid questions, even, whether to walk on the right or on the left side of the promenade. That is why this encephalic wealth was squandered, or rather frittered away, without any use to either himself or the world.

In this outcome, education may step more or less rough-shod, with modifications which altogether alter the program. This, however, not by direct influence of the reason over the will, something which there is not except with a corresponding predisposition of the will, but by early developed and settled *habit* which removes from the entire soul of a human being all accessibility of intellectual means.

> And let me wring your heart, for so I shall,
> If it be made of penetrable stuff;
> If damned custom have not brass'd it so,
> That it be proof and bulwark against sense."—Shakespeare.

That is why, turning to our subject of physiological therapeutics, we must say that it is this only which is promising as remedial measure. Psychological influence must realize itself also as a habit in order to exercise an antagonizing power.

> That monster, custom, who all sense doth eat,
> Of habits devil, is angel yet in this,
> That to the use of actions fair and good
> He likewise gives a frock and livery,
> That aptly is put on."—Shakespeare.

In civilized life there is a good deal of virtue going on quite *machinalement*, where the subject of a full show of social propriety cannot claim ethically a greater merit than to have lent itself to the drilling by its environments:

> "Assume a virtue if you have it not."—Shakespeare.

If the same individual had been born under other circumstances, outwardly, it would have become a member of society which would have to be classified quite differently. This is what makes the law such an equivocal, not to say gruesome, institution, and jurisprudence to the philosophically deeper looking student such a repulsive branch of learning.

Now, *virtue* is a notion to which as such we cannot give a place in physiology. But if we may not marshal it as a physiological factor, we may for all that recognize it physiologically.

And we believe we touch in this item a relation which in psychology has been far too much neglected, and in physiology, for the matter of that, too. This is the bodily and mental equivalence of

Virtue and Health. These two are only different ways of looking at the same condition. One additional circumstance, however, has to be minded in working in this line. This is the abuse which these two words have had to undergo at the hands of antagonistically inclined vocabulators. It is an abuse which continues, if not frauduently, then from lack of philosophical method and skill, or merely from want of independent research. Taking the Pope of Rome and Professor Pettenkofer, of the University of Munich, the former a man of far higher social standing than learning, at least useful learning, the other a man of by far more extensive useful learning than social adequate standing, the ideas of either of health and virtue will turn out considerably different, and similarly there will be fundamental discrepancy if we confront similar other functionaries. The words have to be quite newly substantiated in order to express the pertinent scope of thought doing universally justice to their symbolization of meaning. But without peradventure they have to come out even, and as long as they do not, there is something wrong on the one or the other side.

As to masturbation, we deem it a considerable error to inculpate sensuality in connection with it. I, for one, do not believe that the vice is to be attributed, or can be excused, by an extra degree of sexual competence.

From my observation of boys and older persons of whom I knew that they had a taint of the vice, I must rather infer the contrary. I have reason to believe that the character which carries the danger of the disorder with it is one of weakness and littleness. When there is a boy of strong tendencies, even if his encephalic outfit only should be so; if only he is vigorous and healthy, so that his strong tendencies are not themselves morbid, it is natural that he will be directed in a normal mode—i. e. toward a girl. His immediate aim may be coarse. But it will assume the shape of pure animalism, not run riot in preternatural by-ways. If we said above that virtue has to come out even with health and beauty, or generally, physiology and psychology, there are data of observation which may serve as vouchers of our theory. It is a mistake to think humanitarian idealism the outworking only of exuberant sentiment. Such idealism has certainly its good ground in physiology, as it is an utter impossibility to want to govern the world by thought, and the love feeling is the sensation which substantiates this view; the healthier, the more vigorous and robust a person is, the less the feeling of love will be exploited for merely individual purposes. Love cannot be imagined without generosity, and generosity in love embraces the conception of its consequences in the offspring. This leads, however, precisely to the idealism which erroneously is supposed limited to psychological refinement. We do not know, but doubt whether any refinement ever did anything to promote idealism in love. Refinement may create cranky poetry, but not the sublime love-feeling which is the outgrowth of teeming health. The sexual feeling is the more a healthy feeling the less it is a localized feeling, and consequently the stronger and healthier a boy is, the more he wants satisfaction in a gratification extending all over and through the body, its very soul, as a wholesome sexual congress alone can afford. The very thought of passing by the girls in sexual pursuit is a morbid one; a sturdy, healthy boy does not think of anything of the kind being in existence.

It is claimed by very many physicians, and most by the general practitioner, that masturbation is not an uncommon disorder, but found almost without any exception in all classes of society, with all the boys in school, nor less with very many out of it. Now, this is a terrible charge. If it be true, the very foundation of society, one should say, is rotten, its very core, mentally and bodily, in a state of decay. And the medical testimony goes further than that. The celebrated psychiater, Von Kraft-Ebing, has given us a volume which shows that man, civilized man, has gone way back behind the animal, nay, worse than that, has done so by dint of civilization and in proportion to his advancement in it. If this be true, and we cannot deny it in the face of the witness-array which the medical profession affords, it is an epidemic worse than which the world never has seen, less loud than your plague, scarlatina and smallpox, but more devastating, more fatally undermining, leaving behind not a reliable stock of health, but universal ruin, a crippled totality, mankind turned away from itself toward decomposition.

As to the etiology of this appalling state of affairs, nothing short of mental alienation extending through all humanity meets with the salient points of diagnosis, a germ of corruption being hidden in civilization which must have its origin in an entire misapprehension of life, a total misconstruing of the biological program of man, including his finest actuation, even in art and ethics, in poetry and science, in economics and politics, in short, in all social and private aspirations of human life.

This, now, we believe, indeed, to be the source of the evil; mankind throughout has a wrong conception of its own nature and destination, and thereby runs riot in its endeavor of satisfaction, meeting in it by self-abuse and pursuits of degrading vilification, with the eternal disappointment of gratification, which is the wail of our time and of which the cause is sought at entirely the wrong place.

Since there is a history of man, the common aspiration of all mankind, the universal goal of all ambition, the fascinating allurement of all poetry, the set purpose of all material pursuit, the bewitching fancy of all young imagination, the sum total of all senile wishes, is *the satisfaction, the removal of want.* What was it we read about, and before that were told, as the finest thing in existence, if it were only to be found? A wand with which we can turn all things immediately to what we would wish, so that nothing would remain of all our wishes, but every one of them fulfilled as soon as felt. What is it we read about day in and day out in the newspapers, the magazines, in short, where we turn our eyes? About this same wand, not any more in the shape we dreamed of as little fanciers, but to the same purpose, Gold, Gold, Gold, the talisman of the satisfaction, the removal of want. And the belief in the possibility of satisfying by gold all want is as serious as everybody in good earnest sets about making it.

Yet, if, in drawing from the data of observation the conclusion to which they give occasion, we tame our will, we must admit, in the first instance, that it is altogether an open question whether it is desirable to satisfy our want, especially to the point of a complete elimination, and in the second instance, that it is more than dubious that we possess in gold, or any other purchasing power, a realization of the wand we dreamed of as little children, and were regaled with as fairy tale in the nursery. If we scrutinize the data of observation which are registered in our encephalon, wherever this may occur in localizing specification, we find, as the most important and the most portentous truth, as a truth which is entirely the contrary of the traditional generally held belief, a relation between want and satisfaction which warrants us in none of the maxims observed so far, especially not by the members of the medical profession, the most important position of humanity, for it is obviously they who, in the long run, have to give to society its lasting ethical education.

There is in all philosophy, in all biology, in all empiricism of practical life no thesis less assailable than the thesis, that there is *no health, no enjoyment, no positive contents of life, in short, no individual assertion, without want.* Mind, we do not mean to turn that round. There is want without health. We have morbid want, want which in its very core is disease. But there is no health without want, and if all philosophy of life thus far emphasized the desirability of eliminating want by its satisfaction, this was a suicidal proceeding, killing out precisely that which is necessarily, constitutionally, the fundamental condition of all enjoyment.

"O lyric love, half angel and half bird,
And all a wonder and a wild desire."

We could easily extend this article beyond the bounds of the entire magazine if we were to bring the biological vouchers in this matter. But the pathology of our subject gives us an illustration than more abjectedly convincing none can be found. In the masturbator, more than in any other of the many who have suffered through indulgence in the fallacy of gratification by utter satisfaction of the want of which the human life is the exhibit, we have an example of the jewel which man spurns in his philosophy of life, to exchange for it a glass bead, because this is so much bigger.

To the hugger-mugger dialectician, indeed, as to a coistril a glass bead like a hen's egg, the philosophy can be charming which does not go beyond the rules of a hound or a race-horse, and which above all does not require any excellence of character, for this is the article which most people do not carry in stock, and of that category is the old philosophy. But the wisdom of life which rests on the ground which the brain prepares for the will to get aware of itself through the means of its study, teaches differently. In it it is clear that the destruction of want is not satisfaction of the same. *Want is the main constituent of life.* If we complain of the trouble of want, and are in a frightful hurry to get rid of it, we do not know what we are doing. And this especially the masturbator is not aware of. As the wanton girl which cuts the budding flower to place it at her bosom to wither there, preventing thereby the beauty which nature in due time would have unfolded, so the masturbator, bodily and mentally, destroys the flower of manhood by satisfying the want in which, expanding, it exhibits. And what a satisfaction!

It is now uncounted millions of years, presumably, although in prehistoric times the problem was more easily solved, that man wants to gratify his desire by satisfying his want, and the first man is to be found yet who was awarded the prize of satisfactory accomplishment. Nor will there ever be any one who succeeds, unless he observes a rule which keeps account of the conditions of nature, and this rule is: *Never to gratify want, except in nursing it through its satisfaction.* Want is not a drawback from happy nature. *It is a normal condition of life.* Want must not be fled from, but cultivated. Man must have his want under control. Want does not mean destitution. It means that want without which there is neither health nor enjoyment, neither personality nor the glory of its exhibit in life.

We may not expand here in treating this subject beyond the limits we imposed upon us through the heading, but will say in regard to this that, considering the charge of the medical profession that there is scarcely a single youth exempt from the vice of masturbation, nothing short of an entire veering about of the philosophy of life can promise a remedy, and this remedy, it seems to us, lies in the dropping of the fallacious hope of satisfying the feeling of want ingrained in us by nature as a constituent of our life, by trying to get rid of it. Gratification of want is impossible, except we take the habit of tolerating it, *of enjoying its possession.* Life is want, and if we destroy it as soon as exhibiting we kill life, and the most frightful example is the masturbator who mangles the pretty picture of healthy love, the wonderful charm of budding life, and makes a dung-hill of all—of man, of woman, of child and flower, of imagination and poetry, of humanity and enthusiasm, of the world itself.

